# Supplementary material for: Effectiveness of Palliative Care before Death in Reducing Emergency Care Utilization for Patients with Terminal Cancer and Trends in the Utilization of Palliative Care from 2005–2018
Source: Healthcare (Basel). 2023 Nov 6;11(21):2907. doi: 10.3390/healthcare11212907 (PMC10647500; doi:10.3390/healthcare11212907)
Supplement: Supplementary file 1 [file healthcare-11-02907-s001.zip › healthcare-2645526-supplementary.pdf]

Supplementary Table S1. Characteristics of patients with terminal cancer

Variable	N	%	PC not enrolled n	PC not enrolled %	PC enrolled n	PC enrolled %	χ2 p-value
Total	605126	100.00	365417	60.39	239709	39.61	
Gender							<0.001
Male	380319	62.85	238437	62.69	141882	37.31	
Female	224807	37.15	126980	56.48	97827	43.52	
Age							<0.001
<55 years	111438	18.42	65626	58.89	45812	41.11	
55-64 years	122092	20.18	69102	56.60	52990	43.40	
65-74 years	138623	22.91	83964	60.57	54659	39.43	
75-84 years	157967	26.10	99718	63.13	58249	36.87	
≧85 years	75006	12.40	47007	62.67	27999	37.33	
Education level							<0.001
Illiteracy or elementary school	328769	54.33	209145	63.61	119624	36.39	
Junior high school	113541	18.76	65461	57.65	48080	42.35	
Senior high school	124607	20.59	69630	55.88	54977	44.12	
University or above	38209	6.31	21181	55.43	17028	44.57	
Martial status							<0.001
Unmarried	43235	7.14	25138	58.14	18097	41.86	
Married	385515	63.71	234782	60.90	150733	39.10	
Divorced and Widowed	176376	29.15	105497	59.81	70879	40.19	

Supplementary Table S1. Characteristics of patients with terminal cancer (cont.)

			PC not enrolled		PC enrolled		χ2
Variable	N	%	n	%	n	%	p-value
Monthly salary							<0.001
≦17,280	187919	31.05	112806	60.03	75113	39.97	
17,281-22,800	232626	38.44	147782	63.53	84844	36.47	
22,801-28,800	49294	8.15	26471	53.70	22823	46.30	
28,801-36,300	39310	6.50	22832	58.08	16478	41.92	
36,301-45,800	45272	7.48	26004	57.44	19268	42.56	
45,801-57,800	18389	3.04	10713	58.26	7676	41.74	
≧57,801	32316	5.34	18809	58.20	13507	41.80	
Urbanization degree of residence area							<0.001
Level 1	150306	24.84	88650	58.98	61656	41.02	
Level 2	175104	28.94	102560	58.57	72544	41.43	
Level 3	99203	16.39	59889	60.37	39314	39.63	
Level 4	98648	16.30	62438	63.29	36210	36.71	
Level 5	19430	3.21	12446	64.06	6984	35.94	
Level 6	32750	5.41	20230	61.77	12520	38.23	
Level 7	29685	4.91	19204	64.69	10481	35.31	
CCI							<0.001
0 point	158925	26.26	91574	57.62	67351	42.38	
1 point	162152	26.80	96607	59.58	65545	40.42	
2 points	108298	17.90	65763	60.72	42535	39.28	
3 points	80068	13.23	50007	62.46	30061	37.54	
>3 points	95683	15.81	61466	64.24	34217	35.76	

Supplementary Table S1. Characteristics of patients with terminal cancer (cont.)

			PC not enrolled		PC enrolled		χ2
Variable	N	%	n	%	n	%	p-value
Cancer type							<0.001
Lung	100230	16.56	61635	61.49	38595	38.51	
Liver	100370	16.59	64262	64.03	36108	35.97	
Colorectal	66508	10.99	39817	59.87	26691	40.13	
Breast	25048	4.14	13545	54.08	11503	45.92	
Oral	33823	5.59	19589	57.92	14234	42.08	
Prostate	15536	2.57	10197	65.63	5339	34.37	
Stomach	29437	4.86	17932	60.92	11505	39.08	
Pancreatic	18772	3.10	9404	50.10	9368	49.90	
Esophageal	18600	3.07	11708	62.95	6892	37.05	
Cervical	8482	1.40	5048	59.51	3434	40.49	
Other	77027	12.73	48507	62.97	28520	37.03	
Multiple cancers	111293	18.39	63773	57.30	47520	42.70	
Ownership of medical institution							<0.001
Public	192654	31.84	110277	57.24	82377	42.76	
Non-public	412472	68.16	255140	61.86	157332	38.14	
Level of medial institution							<0.001
Medical center	296872	49.06	174174	58.67	122698	41.33	
Regional hospital	232295	38.39	136389	58.71	95906	41.29	
Local hospital	41438	6.85	30084	72.60	11354	27.40	
Primary clinic	34521	5.70	24770	71.75	9751	28.25	

PC: palliative care CCI: Charlson Comorbidity index

Supplementary Table S2. Comparison of patients with terminal cancer enrolled in palliative care 1 week before death and those not enrolled (before and after PSM)

		Before matching							After 1:1 matching						
				No		Received		χ^2			No		Received		χ^2
**Variable**		N	%	n_0	%	n_1	%	p-value	N	%	n_0	%	n_1	%	p-value
Total		570082	100.00	365417	64.10	204665	35.90		336624	100.00	168312	50.00	168312	50.00	
Sex								<0.001							0.794
	Male	358764	62.93	238437	65.25	120327	58.79		206269	61.28	103097	61.25	103172	61.30	
	Female	211318	37.07	126980	34.75	84338	41.21		130355	38.72	65215	38.75	65140	38.70	
Age								<0.001							0.924
	< 55 years	105323	18.48	65626	17.96	39697	19.40		59423	17.65	29649	17.62	29774	17.69	
	55-64 years	114856	20.15	69102	18.91	45754	22.36		69173	20.55	34562	20.53	34611	20.56	
	65-74 years	130358	22.87	83964	22.98	46394	22.67		75897	22.55	37952	22.55	37945	22.54	
	75-84 years	148748	26.09	99718	27.29	49030	23.96		87965	26.13	43984	26.13	43981	26.13	
	≧85 years	70797	12.42	47007	12.86	23790	11.62		44166	13.12	22165	13.17	22001	13.07	
CCI								<0.001							0.995
	0 point	149599	26.24	91574	25.06	58025	28.35		90702	26.94	45334	26.93	45368	26.95	
	1 point	152672	26.78	96607	26.44	56065	27.39		90774	26.97	45360	26.95	45414	26.98	
	2 points	102306	17.95	65763	18.00	36543	17.86		61126	18.16	30610	18.19	30516	18.13	
	3 points	75183	13.19	50007	13.68	25176	12.30		43286	12.86	21633	12.85	21653	12.86	
	>3 points	90322	15.84	61466	16.82	28856	14.10		50736	15.07	25375	15.08	25361	15.07	
Cancer type								<0.001							1.000
	Lung	94526	16.58	61635	16.87	32891	16.07		54991	16.34	27484	16.33	27507	16.34	
	Liver	93234	16.35	64262	17.59	28972	14.16		50546	15.02	25248	15.00	25298	15.03	
	Colorectal	62721	11.00	39817	10.90	22904	11.19		38016	11.29	19070	11.33	18946	11.26	
	Breast	23271	4.08	13545	3.71	9726	4.75		14352	4.26	7184	4.27	7168	4.26	

Oral	32256	5.66	19589	5.36	12667	6.19	20251	6.02	10132	6.02	10119	6.01
Prostate	14774	2.59	10197	2.79	4577	2.24	8678	2.58	4360	2.59	4318	2.57
Stomach	27798	4.88	17932	4.91	9866	4.82	15911	4.73	7910	4.70	8001	4.75
Pancreatic	17369	3.05	9404	2.57	7965	3.89	10489	3.12	5252	3.12	5237	3.11
Esophageal	17700	3.10	11708	3.20	5992	2.93	9811	2.91	4914	2.92	4897	2.91
Cervical	8078	1.42	5048	1.38	3030	1.48	4581	1.36	2300	1.37	2281	1.36
Other	73248	12.85	48507	13.27	24741	12.09	41663	12.38	20811	12.36	20852	12.39
Multiple cancers	105107	18.44	63773	17.45	41334	20.20	67335	20.00	33647	19.99	33688	20.02

PSM, propensity score matching

CCI, Charlson Comorbidity Index

Supplementary Table S3. Comparison of patients with terminal cancer enrolled in palliative care 4 week before death and those not enrolled (before and after PSM)

	Before matching							After 1:1 matching						
			No		Received		χ^2			No		Received		χ^2
**Variable**	N	%	n_0	%	n_1	%	p-value	N	%	n_0	%	n_1	%	p-value
Total	480212	100.00	365417	76.09	114795	23.91		217080	100.00	108540	50.00	108540	50.00	
Sex							<0.001							0.724
Male	304056	63.32	238437	65.25	65619	57.16		127692	58.82	63805	58.78	63887	58.86	
Female	176156	36.68	126980	34.75	49176	42.84		89388	41.18	44735	41.22	44653	41.14	
Age							<0.001							0.952
< 55 years	88959	18.52	65626	17.96	23333	20.33		41485	19.11	20690	19.06	20795	19.16	
55-64 years	94922	19.77	69102	18.91	25820	22.49		47371	21.82	23677	21.81	23694	21.83	
65-74 years	109290	22.76	83964	22.98	25326	22.06		48489	22.34	24243	22.34	24246	22.34	
75-84 years	126573	26.36	99718	27.29	26855	23.39		53016	24.42	26518	24.43	26498	24.41	
≧85 years	60468	12.59	47007	12.86	13461	11.73		26719	12.31	13412	12.36	13307	12.26	
CCI							<0.001							1.000
0 point	125062	26.04	91574	25.06	33488	29.17		61877	28.50	30917	28.48	30960	28.52	
1 point	128342	26.73	96607	26.44	31735	27.64		59616	27.46	29828	27.48	29788	27.44	
2 points	86443	18.00	65763	18.00	20680	18.01		39393	18.15	19696	18.15	19697	18.15	
3 points	63546	13.23	50007	13.68	13539	11.79		26158	12.05	13077	12.05	13081	12.05	
>3 points	76819	16.00	61466	16.82	15353	13.37		30036	13.84	15022	13.84	15014	13.83	
Cancer type							<0.001							1.000
Lung	80258	16.71	61635	16.87	18623	16.22		35708	16.45	17832	16.43	17876	16.47	
Liver	77214	16.08	64262	17.59	12952	11.28		25730	11.85	12867	11.85	12863	11.85	
Colorectal	53093	11.06	39817	10.90	13276	11.56		24829	11.44	12413	11.44	12416	11.44	
Breast	19235	4.01	13545	3.71	5690	4.96		10145	4.67	5085	4.68	5060	4.66	

Oral	27783	5.79	19589	5.36	8194	7.14	15123	6.97	7565	6.97	7558	6.96
Prostate	13024	2.71	10197	2.79	2827	2.46	5528	2.55	2765	2.55	2763	2.55
Stomach	23287	4.85	17932	4.91	5355	4.66	9831	4.53	4903	4.52	4928	4.54
Pancreatic	13504	2.81	9404	2.57	4100	3.57	6941	3.20	3460	3.19	3481	3.21
Esophageal	15143	3.15	11708	3.20	3435	2.99	6521	3.00	3256	3.00	3265	3.01
Cervical	6922	1.44	5048	1.38	1874	1.63	3205	1.48	1612	1.49	1593	1.47
Other	63103	13.14	48507	13.27	14596	12.71	27653	12.74	13831	12.74	13822	12.73
Multiple cancers	87646	18.25	63773	17.45	23873	20.80	45866	21.13	22951	21.15	22915	21.11

PSM, propensity score matching

CCI, Charlson Comorbidity Index

Supplementary Table S4. Comparison of patients with terminal cancer enrolled in palliative care 8 week before death and those not enrolled (before and after PSM)

		Before matching							After 1:1 matching						
				No		Received		χ^2			No		Received		χ^2
**Variable**		N	%	n_0	%	n_1	%	p-value	N	%	n_0	%	n_1	%	p-value
Total		427710	100.00	365417	85.44	62293	14.56		123500	100.00	61750	50.00	61750	50.00	
Sex								<0.001							0.986
	Male	273000	63.83	238437	65.25	34563	55.48		68962	55.84	34483	55.84	34479	55.84	
	Female	154710	36.17	126980	34.75	27730	44.52		54538	44.16	27267	44.16	27271	44.16	
Age								<0.001							1.000
	< 55 years	78331	18.31	65626	17.96	12705	20.40		24810	20.09	12393	20.07	12417	20.11	
	55-64 years	82960	19.40	69102	18.91	13858	22.25		27413	22.20	13717	22.21	13696	22.18	
	65-74 years	97369	22.77	83964	22.98	13405	21.52		26696	21.62	13344	21.61	13352	21.62	
	75-84 years	114232	26.71	99718	27.29	14514	23.30		29001	23.48	14505	23.49	14496	23.48	
	≧85 years	54818	12.82	47007	12.86	7811	12.54		15580	12.62	7791	12.62	7789	12.61	
CCI								<0.001							1.000
	0 point	109595	25.62	91574	25.06	18021	28.93		35805	28.99	17895	28.98	17910	29.00	
	1 point	113649	26.57	96607	26.44	17042	27.36		33709	27.29	16854	27.29	16855	27.30	
	2 points	77274	18.07	65763	18.00	11511	18.48		22750	18.42	11366	18.41	11384	18.44	
	3 points	57320	13.40	50007	13.68	7313	11.74		14506	11.75	7262	11.76	7244	11.73	
	>3 points	69872	16.34	61466	16.82	8406	13.49		16730	13.55	8373	13.56	8357	13.53	
Cancer type								<0.001							1.000
	Lung	71860	16.80	61635	16.87	10225	16.41		20353	16.48	10176	16.48	10177	16.48	
	Liver	70240	16.42	64262	17.59	5978	9.60		11949	9.68	5976	9.68	5973	9.67	
	Colorectal	47225	11.04	39817	10.90	7408	11.89		14630	11.85	7307	11.83	7323	11.86	
	Breast	17046	3.99	13545	3.71	3501	5.62		6917	5.60	3457	5.60	3460	5.60	

Oral	24270	5.67	19589	5.36	4681	7.51		9276	7.51	4642	7.52	4634	7.50
Prostate	11997	2.80	10197	2.79	1800	2.89		3571	2.89	1785	2.89	1786	2.89
Stomach	20574	4.81	17932	4.91	2642	4.24		5139	4.16	2570	4.16	2569	4.16
Pancreatic	11216	2.62	9404	2.57	1812	2.91		3499	2.83	1752	2.84	1747	2.83
Esophageal	13442	3.14	11708	3.20	1734	2.78		3396	2.75	1692	2.74	1704	2.76
Cervical	6146	1.44	5048	1.38	1098	1.76		2066	1.67	1025	1.66	1041	1.69
Other	56704	13.26	48507	13.27	8197	13.16		16268	13.17	8140	13.18	8128	13.16
Multiple cancers	76990	18.00	63773	17.45	13217	21.22		26436	21.41	13228	21.42	13208	21.39

PSM, propensity score matching

CCI, Charlson Comorbidity Index

Supplementary Table S5. Comparison of patients with terminal cancer enrolled in palliative care 12 week before death and those not enrolled (before and after PSM)

		Before matching							After 1:1 matching						
				No		Received		χ^2			No		Received		χ^2
**Variable**		N	%	n_0	%	n_1	%	p-value	N	%	n_0	%	n_1	%	p-value
Total		405838	100.00	365417	90.04	40421	9.96		80618	100.00	40309	50.00	40309	50.00	
Sex								<0.001							0.927
	Male	260481	64.18	238437	65.25	22044	54.54		44064	54.66	22039	54.68	22025	54.64	
	Female	145357	35.82	126980	34.75	18377	45.46		36554	45.34	18270	45.32	18284	45.36	
Age								<0.001							1.000
	< 55 years	73713	18.16	65626	17.96	8087	20.01		16033	19.89	8011	19.87	8022	19.90	
	55-64 years	77882	19.19	69102	18.91	8780	21.72		17529	21.74	8764	21.74	8765	21.74	
	65-74 years	92551	22.80	83964	22.98	8587	21.24		17168	21.30	8589	21.31	8579	21.28	
	75-84 years	109250	26.92	99718	27.29	9532	23.58		19056	23.64	9530	23.64	9526	23.63	
	≧85 years	52442	12.92	47007	12.86	5435	13.45		10832	13.44	5415	13.43	5417	13.44	
CCI								<0.001							1.000
	0 point	103065	25.40	91574	25.06	11491	28.43		22963	28.48	11479	28.48	11484	28.49	
	1 point	107555	26.50	96607	26.44	10948	27.08		21813	27.06	10905	27.05	10908	27.06	
	2 points	73381	18.08	65763	18.00	7618	18.85		15172	18.82	7583	18.81	7589	18.83	
	3 points	54766	13.49	50007	13.68	4759	11.77		9474	11.75	4734	11.74	4740	11.76	
	>3 points	67071	16.53	61466	16.82	5605	13.87		11196	13.89	5608	13.91	5588	13.86	
Cancer type								<0.001							1.000
	Lung	68445	16.87	61635	16.87	6810	16.85		13610	16.88	6806	16.88	6804	16.88	
	Liver	67879	16.73	64262	17.59	3617	8.95		7232	8.97	3616	8.97	3616	8.97	
	Colorectal	44675	11.01	39817	10.90	4858	12.02		9700	12.03	4855	12.04	4845	12.02	
	Breast	16097	3.97	13545	3.71	2552	6.31		5096	6.32	2549	6.32	2547	6.32	

Oral	22515	5.55	19589	5.36	2926	7.24		5816	7.21	2911	7.22	2905	7.21
Prostate	11516	2.84	10197	2.79	1319	3.26		2625	3.26	1313	3.26	1312	3.25
Stomach	19537	4.81	17932	4.91	1605	3.97		3176	3.94	1584	3.93	1592	3.95
Pancreatic	10405	2.56	9404	2.57	1001	2.48		1964	2.44	981	2.43	983	2.44
Esophageal	12729	3.14	11708	3.20	1021	2.53		1995	2.47	996	2.47	999	2.48
Cervical	5750	1.42	5048	1.38	702	1.74		1380	1.71	682	1.69	698	1.73
Other	53855	13.27	48507	13.27	5348	13.23		10695	13.27	5347	13.27	5348	13.27
Multiple cancers	72435	17.85	63773	17.45	8662	21.43		17329	21.50	8669	21.51	8660	21.48

PSM, propensity score matching

CCI, Charlson Comorbidity Index

Supplementary Table S6. Comparison of patients with terminal cancer enrolled in palliative care 24 week before death and those not enrolled (before and after PSM)

	Before matching							After 1:1 matching						
			No		Received		χ^2			No		Received		χ^2
**Variable**	N	%	n_0	%	n_1	%	p-value	N	%	n_0	%	n_1	%	p-value
Total	383244	100.00	365417	95.35	17827	4.65		35622	100.00	17811	50.00	17811	50.00	
Sex							<0.001							0.907
Male	247811	64.66	238437	65.25	9374	52.58		18758	52.66	9385	52.69	9373	52.62	
Female	135433	35.34	126980	34.75	8453	47.42		16864	47.34	8426	47.31	8438	47.38	
Age							<0.001							1.000
< 55 years	68907	17.98	65626	17.96	3281	18.40		6543	18.37	3268	18.35	3275	18.39	
55-64 years	72949	19.03	69102	18.91	3847	21.58		7683	21.57	3838	21.55	3845	21.59	
65-74 years	87731	22.89	83964	22.98	3767	21.13		7535	21.15	3768	21.16	3767	21.15	
75-84 years	103983	27.13	99718	27.29	4265	23.92		8532	23.95	4268	23.96	4264	23.94	
≧85 years	49674	12.96	47007	12.86	2667	14.96		5329	14.96	2669	14.99	2660	14.93	
CCI							<0.001							1.000
0 point	96413	25.16	91574	25.06	4839	27.14		9678	27.17	4839	27.17	4839	27.17	
1 point	101352	26.45	96607	26.44	4745	26.62		9488	26.64	4747	26.65	4741	26.62	
2 points	69194	18.05	65763	18.00	3431	19.25		6854	19.24	3427	19.24	3427	19.24	
3 points	52183	13.62	50007	13.68	2176	12.21		4337	12.18	2167	12.17	2170	12.18	
>3 points	64102	16.73	61466	16.82	2636	14.79		5265	14.78	2631	14.77	2634	14.79	
Cancer type							<0.001							1.000
Lung	64691	16.88	61635	16.87	3056	17.14		6109	17.15	3053	17.14	3056	17.16	
Liver	65713	17.15	64262	17.59	1451	8.14		2902	8.15	1451	8.15	1451	8.15	
Colorectal	41978	10.95	39817	10.90	2161	12.12		4326	12.14	2167	12.17	2159	12.12	
Breast	14946	3.90	13545	3.71	1401	7.86		2805	7.87	1404	7.88	1401	7.87	

Oral	20720	5.41	19589	5.36	1131	6.34	2253	6.32	1126	6.32	1127	6.33
Prostate	10901	2.84	10197	2.79	704	3.95	1411	3.96	707	3.97	704	3.95
Stomach	18537	4.84	17932	4.91	605	3.39	1209	3.39	604	3.39	605	3.40
Pancreatic	9718	2.54	9404	2.57	314	1.76	622	1.75	308	1.73	314	1.76
Esophageal	12091	3.15	11708	3.20	383	2.15	750	2.11	373	2.09	377	2.12
Cervical	5374	1.40	5048	1.38	326	1.83	642	1.80	320	1.80	322	1.81
Other	50876	13.28	48507	13.27	2369	13.29	4740	13.31	2371	13.31	2369	13.30
Multiple cancers	67699	17.66	63773	17.45	3926	22.02	7853	22.05	3927	22.05	3926	22.04

PSM, propensity score matching

CCI, Charlson Comorbidity Index

Supplementary Table S7. Differences in emergency care utilization and CPR according to year among patients with terminal cancer enrolled in palliative care at 1, 4, 8, 12, and 24 weeks before death and those not enrolled

	1 week before death			4 weeks before death			8 weeks before death			12 weeks before death			24 weeks before death		
	No	Received		No	Received		No	Received		No	Received		No	Received	
	Use%[a]	Use%[b]	b/a	Use%[a]	Use%[b]	b/a	Use%[a]	Use%[b]	b/a	Use%[a]	Use%[b]	b/a	Use%[a]	Use%[b]	b/a
Emergency															
Year of death															
2005	20.83	10.69	0.51	44.72	26.97	0.60	59.06	40.43	0.68	63.31	50.14	0.79	68.49	65.07	0.95
2006	21.63	9.91	0.46	45.16	27.51	0.61	59.45	43.37	0.73	65.68	52.07	0.79	75.38	63.53	0.84
2007	21.82	11.69	0.54	45.53	29.93	0.66	59.30	44.97	0.76	66.56	55.10	0.83	71.97	70.23	0.98
2008	22.93	11.89	0.52	44.87	29.92	0.67	59.33	43.39	0.73	66.18	51.15	0.77	75.75	65.12	0.86
2009	22.63	11.67	0.52	47.25	30.62	0.65	59.75	46.04	0.77	66.19	52.97	0.80	74.69	65.16	0.87
2010	24.96	12.69	0.51	51.54	32.90	0.64	66.01	48.86	0.74	71.38	58.48	0.82	81.88	68.12	0.83
2011	27.59	12.59	0.46	53.98	33.27	0.62	66.98	49.55	0.74	71.79	58.48	0.81	80.88	69.22	0.86
2012	30.58	13.02	0.43	57.02	34.91	0.61	69.06	51.89	0.75	74.30	62.95	0.85	82.02	73.45	0.90
2013	31.28	12.97	0.41	57.77	35.40	0.61	69.80	53.16	0.76	75.37	62.57	0.83	81.10	72.59	0.90
2014	33.37	13.34	0.40	56.86	35.55	0.63	67.88	53.08	0.78	72.95	63.26	0.87	78.97	73.83	0.93
2015	34.33	14.30	0.42	59.00	37.16	0.63	69.36	54.40	0.78	73.80	64.38	0.87	79.26	75.20	0.95
2016	34.50	14.22	0.41	58.57	36.94	0.63	69.03	54.88	0.80	74.04	64.15	0.87	80.41	74.77	0.93
2017	34.55	13.80	0.40	57.57	36.86	0.64	67.65	53.37	0.79	72.08	61.90	0.86	78.09	73.78	0.94
2018	31.86	13.34	0.42	56.58	36.37	0.64	67.41	52.81	0.78	73.15	61.26	0.84	79.56	71.71	0.90
CPR															
Year of death															
2005	13.82	1.95	0.14	14.31	2.26	0.16	14.38	2.89	0.20	15.41	2.94	0.19	15.41	3.08	0.20
2006	13.30	1.70	0.13	13.53	2.37	0.18	14.31	3.26	0.23	13.49	3.31	0.25	12.16	5.17	0.43

2007	14.51	1.88	0.13	14.87	2.35	0.16	14.73	3.29	0.22	13.88	4.00	0.29	16.18	5.49	0.34
2008	12.70	1.35	0.11	13.49	2.15	0.16	14.74	3.16	0.21	14.82	3.97	0.27	15.80	5.99	0.38
2009	11.94	1.25	0.10	11.95	1.63	0.14	10.96	2.08	0.19	11.52	2.55	0.22	9.77	3.76	0.38
2010	10.80	1.06	0.10	11.26	1.40	0.12	11.74	2.07	0.18	12.28	3.09	0.25	14.25	4.83	0.34
2011	11.30	1.34	0.12	11.93	1.94	0.16	12.19	2.34	0.19	12.11	2.73	0.23	11.09	2.49	0.22
2012	11.23	1.54	0.14	11.80	2.10	0.18	12.05	2.79	0.23	11.72	3.24	0.28	11.43	3.57	0.31
2013	11.45	1.77	0.15	11.70	2.12	0.18	12.16	2.59	0.21	11.66	2.92	0.25	10.88	3.93	0.36
2014	11.95	1.91	0.16	12.69	2.27	0.18	12.87	2.60	0.20	13.60	3.06	0.23	13.48	3.97	0.29
2015	12.66	2.03	0.16	13.20	2.27	0.17	13.49	2.57	0.19	13.80	2.71	0.20	14.90	3.77	0.25
2016	12.61	2.15	0.17	13.46	2.53	0.19	13.89	3.04	0.22	13.69	3.27	0.24	13.28	4.35	0.33
2017	12.45	1.95	0.16	12.87	2.23	0.17	12.76	2.44	0.19	12.60	2.76	0.22	12.72	3.16	0.25
2018	11.98	1.80	0.15	12.56	2.07	0.16	12.68	2.54	0.20	12.85	2.82	0.22	13.25	3.22	0.24

CRP, cardiopulmonary resuscitation

Supplementary Table S8. Differences in endotracheal intubation and ICU admission according to year among patients with terminal cancer enrolled in palliative care at 1, 4, 8, 12, and 24 weeks before death and those not enrolled

	1 week before death			4 weeks before death			8 weeks before death			12 weeks before death			24 weeks before death		
	No	Received		No	Received		No	Received		No	Received		No	Received	
	Use%[a]	Use%[b]	b/a	Use%[a]	Use%[b]	b/a	Use%[a]	Use%[b]	b/a	Use%[a]	Use%[b]	b/a	Use%[a]	Use%[b]	b/a
ET															
Year of death															
2005	26.38	2.97	0.11	27.97	4.13	0.15	29.53	5.79	0.20	30.95	7.00	0.23	29.11	9.93	0.34
2006	26.13	2.29	0.09	28.07	3.82	0.14	29.36	6.00	0.20	31.36	7.57	0.24	32.83	11.85	0.36
2007	25.92	2.45	0.09	27.91	3.51	0.13	30.11	5.26	0.17	30.70	6.20	0.20	37.28	8.38	0.22
2008	23.31	2.00	0.09	24.41	3.42	0.14	26.44	5.25	0.20	28.18	6.89	0.24	31.88	10.63	0.33
2009	22.91	1.68	0.07	24.43	2.63	0.11	24.56	4.27	0.17	24.55	5.85	0.24	22.81	9.77	0.43
2010	21.06	1.40	0.07	22.55	2.25	0.10	25.23	3.03	0.12	26.50	3.80	0.14	28.99	6.28	0.22
2011	20.02	2.31	0.12	22.19	3.36	0.15	23.77	3.92	0.16	24.42	4.13	0.17	27.15	5.35	0.20
2012	19.87	3.17	0.16	21.73	4.30	0.20	23.06	5.72	0.25	23.73	6.68	0.28	25.24	7.62	0.30
2013	20.73	4.01	0.19	21.80	4.77	0.22	23.62	5.59	0.24	24.03	6.31	0.26	24.71	7.77	0.31
2014	20.28	4.48	0.22	22.24	4.85	0.22	24.34	5.41	0.22	25.43	6.43	0.25	26.04	8.40	0.32
2015	20.67	5.28	0.26	22.61	5.68	0.25	24.04	5.90	0.25	24.93	6.50	0.26	26.08	8.19	0.31
2016	20.61	5.55	0.27	22.55	5.95	0.26	23.93	6.19	0.26	24.24	6.43	0.27	24.05	7.78	0.32
2017	20.14	5.40	0.27	21.85	5.63	0.26	22.88	5.57	0.24	23.15	6.37	0.28	23.77	7.79	0.33
2018	19.25	5.38	0.28	20.99	5.83	0.28	22.35	6.06	0.27	23.00	6.58	0.29	23.98	8.42	0.35
ICU															
Year of death															
2005	26.11	2.46	0.09	28.23	4.31	0.15	29.87	6.81	0.23	32.07	7.84	0.24	36.64	11.64	0.32
2006	29.75	2.71	0.09	30.92	5.49	0.18	33.36	8.67	0.26	34.56	11.01	0.32	39.51	17.02	0.43

2007	28.41	2.54	0.09	30.73	4.44	0.14	33.07	6.90	0.21	35.33	8.62	0.24	41.33	13.29	0.32
2008	26.79	2.72	0.10	28.44	4.75	0.17	30.93	7.34	0.24	32.88	10.33	0.31	37.87	16.08	0.42
2009	28.92	2.26	0.08	30.79	4.15	0.13	33.16	6.58	0.20	32.77	7.65	0.23	33.58	13.28	0.40
2010	26.60	1.94	0.07	29.10	3.62	0.12	32.08	5.68	0.18	35.07	7.60	0.22	39.86	9.42	0.24
2011	26.59	4.52	0.17	28.48	6.30	0.22	30.30	7.81	0.26	33.07	8.52	0.26	38.43	11.85	0.31
2012	27.20	6.88	0.25	29.37	9.17	0.31	31.47	11.25	0.36	33.44	12.70	0.38	37.02	15.60	0.42
2013	28.00	9.48	0.34	29.67	11.31	0.38	32.21	12.51	0.39	33.91	15.12	0.45	36.17	16.78	0.46
2014	27.14	10.96	0.40	28.81	12.68	0.44	32.18	14.02	0.44	33.98	16.02	0.47	36.78	18.68	0.51
2015	26.98	12.42	0.46	28.69	13.91	0.48	31.02	15.39	0.50	32.88	17.02	0.52	36.32	21.27	0.59
2016	26.20	13.53	0.52	28.62	14.87	0.52	31.34	15.32	0.49	32.35	16.47	0.51	35.47	18.92	0.53
2017	26.95	13.65	0.51	29.21	15.04	0.51	30.98	16.83	0.54	32.25	18.37	0.57	35.68	22.84	0.64
2018	26.03	14.08	0.54	28.26	15.40	0.54	31.18	17.08	0.55	32.57	19.08	0.59	36.23	23.38	0.65

ET, endotracheal intubation

ICU, intensive care unit
